# Mechanisms of UV-induced mutations and skin cancer

**DOI:** 10.1007/s42764-020-00009-8

**Published:** 2020-03-19

**Authors:** Gerd P. Pfeifer

**Affiliations:** Center for Epigenetics, Van Andel Institute, Grand Rapids, MI 49503 USA

**Keywords:** Ultraviolet, UV, Cyclobutane pyrimidine dimer, (6–4) Photoproduct, Mutations, DNA repair, Skin cancer, Melanoma

## Abstract

Ultraviolet (UV) irradiation causes various types of DNA damage, which leads to specific mutations and the emergence of skin cancer in humans, often decades after initial exposure. Different UV wavelengths cause the formation of prominent UV-induced DNA lesions. Most of these lesions are removed by the nucleotide excision repair pathway, which is defective in rare genetic skin disorders referred to as xeroderma pigmentosum. A major role in inducing sunlight-dependent skin cancer mutations is assigned to the cyclobutane pyrimidine dimers (CPDs). In this review, we discuss the mechanisms of UV damage induction, the genomic distribution of this damage, relevant DNA repair mechanisms, the proposed mechanisms of how UV-induced CPDs bring about DNA replication-dependent mutagenicity in mammalian cells, and the strong signature of UV damage and mutagenesis found in skin cancer genomes.

## Introduction

Ultraviolet light is a type of electromagnetic radiation invisible to the human eye. This radiation is abundantly present in the electromagnetic waves emitted by the sun. The ultraviolet light spectrum is conventionally subdivided into UVA radiation (320–400 nm), UVB radiation (280–320 nm) and UVC radiation (100–280 nm) although slightly different definitions also exist (i.e. 280–315 nm for the UVB range, and 315–400 nm for UVA). Wavelengths below 200 nm are effectively absorbed and eliminated by oxygen in the earth’s atmosphere. The UVC spectrum below 280 nm as well as most of the radiation between 280 and 310 nm are strongly absorbed by stratospheric ozone. However, there is a fraction of the UVB radiation that does reach the surface of the planet and can cause DNA damage in exposed tissue. This fraction of solar UV radiation is most relevant for human health and consists of wavelengths of approximately 300–320 nm.

Sunlight exposure has long been linked to skin cancer in many epidemiological studies. This association has been most obvious for non-melanoma skin cancers (basal and squamous cell carcinomas) (Greinert 2009; Leiter et al. 2014). For melanoma risk, an involvement of sunlight is also apparent in many cases (Carr et al. 2020). The cells of origin for these tumors are thought to be epidermal keratinocytes for non-melanoma skin tumors and melanocytes or their precursors for melanoma. Non-melanoma skin cancer is a very common type of tumor diagnosed often in fair-skinned populations that reside in geographic areas of high sun exposure. The incidence of both non-melanoma and melanoma skin cancers has risen substantially in recent times (Houghton and Polsky 2002; Donaldson and Coldiron 2011; Leiter et al. 2014). While it is straightforward and curative in most cases to surgically remove basal and squamous cell carcinomas, melanomas are more difficult to treat, in particular when identified late, because of the tendency of these tumors to metastasize to different organs. Although treatment strategies based on inhibiting the BRAF kinase in melanomas have shown initial promise (Luke et al. 2017), resistance to this treatment develops almost invariably.

Epidemiological evidence clearly points to solar ultraviolet radiation as an overwhelming cause of skin cancer (Tucker 2008; Greinert 2009; Leiter et al. 2014). Because the DNA damaging agent is well known, it is possible to mechanistically dissect the various pathways that lead to the formation of the damage, even genome-wide, to understand the DNA repair pathways that reverse or mitigate the damage, as well as the processes that occur before and during DNA replication that will eventually lead to the mutations that characterize skin cancer genomes.

## UV-induced DNA damage formation

**Fig. 1:**
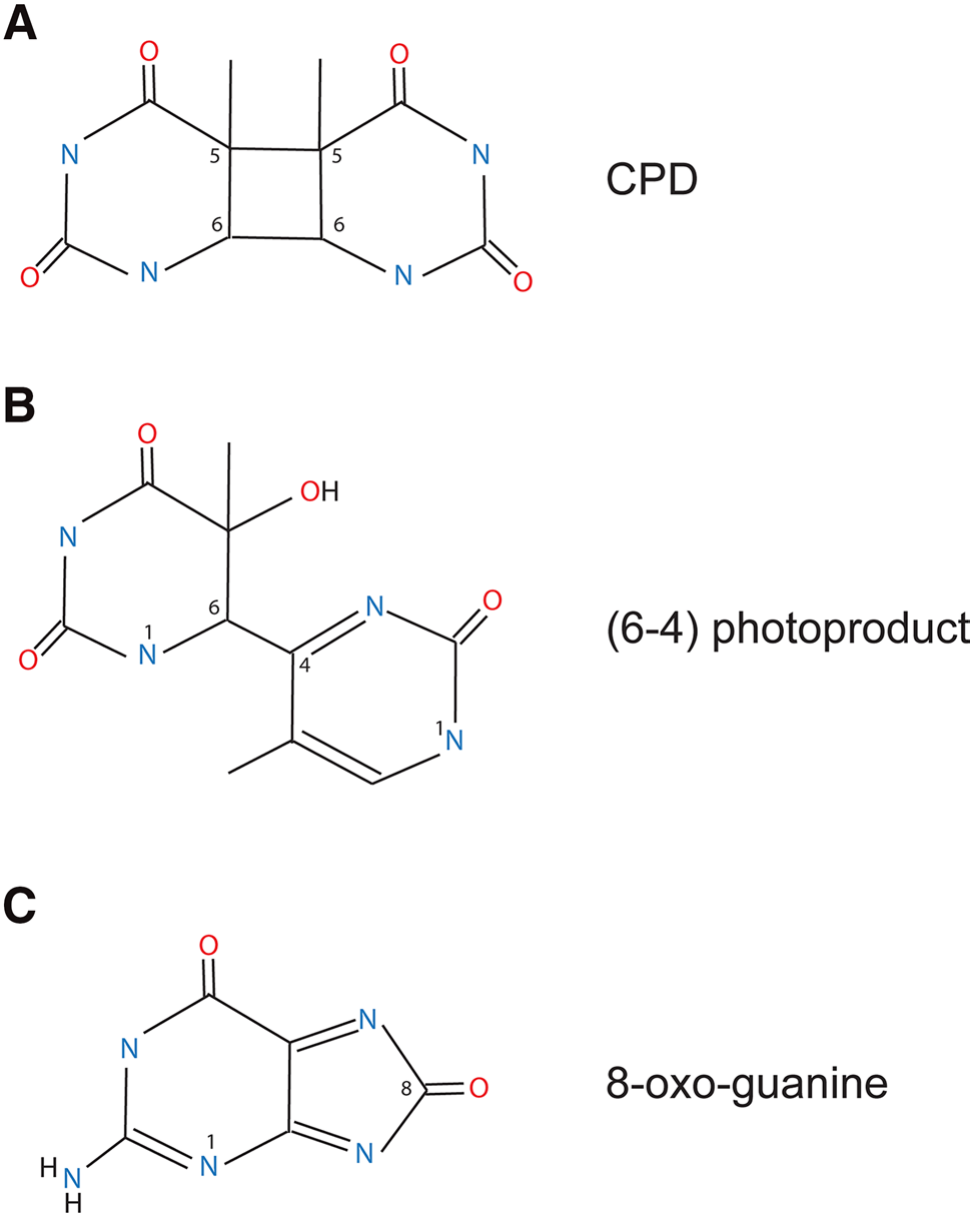
The major DNA damage products induced by solar UVB irradiation. **a** Cyclobutane pyrimidine dimer (CPD) at TT sequences. **b** Pyrimidine (6–4) pyrimidone photoproduct [(6–4)PP] at TT sequences. **c** 8-Oxoguanine

The damage to DNA caused by UV radiation can be direct or indirect. The direct DNA damage consists predominantly of dimerized pyrimidines, and it is this type of damage that is likely the most relevant for skin cancer induction. The dimerization of two adjacent pyrimidines in DNA by ultraviolet light has been known for about 60 years (Beukers et al. 2008). This dimer formation is dependent on the UV absorption of the DNA bases and occurs preferentially in the UVB and UVC range through the formation of electronic excited states (Markovitsi 2016). For example, germicidal lamps emitting at a wavelength of 254 nm effectively induce dimerized pyrimidines. However, this event still is quite effective in the UVB range (280–320 nm). The dimerization involves the formation of *cis-syn* cyclobutane pyrimidine dimers (CPDs) as the most prominent lesion in double-stranded DNA, or of pyrimidine (6–4) pyrimidone photoproducts [(6–4) photoproducts; (6–4)PPs] as the second most frequent DNA lesion in the UVB range (Pfeifer 1997). The structures of these DNA lesions are shown in Fig. 1. In addition to these dipyrimidine lesions, some other DNA photoproducts can form, including, for example, pyrimidine photohydrates and rare lesions involving purine dimers (Pfeifer 1997).

In cyclobutane pyrimidine dimers, a cyclobutane ring is formed that involves the C5, C6 double bonds of the two adjacent pyrimidines (thymine, cytosine, or the cytosine derivative 5-methylcytosine and its oxidation products) (Fig. 1a). The formation of the (6–4) photoproducts involves a rearrangement through an oxetane intermediate and leads to the creation of a stable bond connecting positions 6 and 4 of the neighboring pyrimidines (Fig. 1b). Depending on the wavelength and on local genome parameters such as sequence specificity and chromatin environment, (6–4) PPs are overall considerably less frequent than CPDs and can be present at only a few percent of the total CPD levels (Mitchell and Nairn 1989; Yoon et al. 2000a, b). UVA radiation between 320 and 400 nm can also induce CPDs in the genome (Douki et al. 2003; Rochette et al. 2003; Ikehata et al. 2008), but a very high dose of UVA (tens of thousands of kJ/m^2^) is required to achieve substantial levels of this type of DNA damage (Besaratinia et al. 2005). Interestingly, only small quantities of (6–4) photoproducts form at wavelengths between 300 and 305 nm and this level further diminishes at longer wavelengths (Besaratinia et al. 2011). A fraction of the (6–4)PPs can be converted to their Dewar valence isomers by the absorption of a photon in the 320 nm range leading to the formation of a lesion that is potentially more mutagenic than its parent product (Douki and Sage 2016). The sunlight wavelengths that reach the earth’s surface are strongly diminished below 300 nm. CPDs are the major DNA damage product in simulated sunlight (UVA + UVB)-irradiated DNA or cells (Sage 1993; Yoon et al. 2000a, b; You et al. 2001; Cadet et al. 2012). Their levels clearly exceed the levels of (6–4)PPs or other lesions. Even though DNA absorption spectra peak at about 260 nm, substantial levels of CPDs are still produced at wavelengths between 300 and 320 nm (Besaratinia et al. 2011).

In addition to pyrimidine dimers, UVA, and also UVB, indirectly can promote the formation of oxidized DNA base damage, for example, in the form of 8-oxo-7,8-dihydro-2′-deoxyguanosine (8-oxo-dG) (Fig. 1C) (Cadet et al. 1997,2015; Kielbassa et al. 1997; Zhang et al. 1997; Kvam and Tyrell 1997; Kuluncsics et al. 1999; Besaratinia et al. 2005). This oxidative damage is often mediated by chromophore-induced photosensitization reactions (Schuch et al. 2017). UVA-mediated genomic toxicity is particularly relevant in certain unnatural situations. For example, the frequent use of indoor tanning beds by populations residing in Northern latitudes can lead to high cumulative doses of UVA radiation (Sample and He 2018).

Taken together, the accumulated data suggest that CPDs are the major mutagenic DNA lesions produced by terrestrial sunlight in the skin (Ikehata et al. 2020).

## Mechanisms of UV mutagenesis

Although the high frequency of cytosine to thymine transition mutations at dipyrimidine sequences has been known for a long time, the precise mechanism how the UV photoproducts forming at these sequences induce such typical mutations is still unknown (Pfeifer et al. 2005). In mammalian cells, the CPD is by far more mutagenic than the (6–4) photoproducts as determined by quantitative mutation reporter experiments done after removal of one or the other type of photoproduct using either CPD-specific- or (6–4)PP-specific DNA photolyases, which revert the specific lesions (You et al. 2001). The explanation for the higher potency of CPDs to induce mutations may lie in several factors including (i) the higher level of induction of CPDs by UVB or sunlight, (ii) the slower repair of CPDs relative to the (6–4) photoproducts (Pfeifer et al. 2005; Mitchell and Fernandez 2011), and (iii) the higher propensity of CPDs to undergo mutagenic bypass by DNA polymerases during DNA replication. However, notwithstanding these findings and assumptions, the exact contribution of (6–4)PPs to UV mutagenesis remains poorly defined (Mitchell and Nairn 1989; Pfeifer 1997; Mitchell and Fernandez 2011).

CPDs form at high frequency at 5′TT dinucleotides. However, UVB-irradiated cells rarely accumulate mutations at 5′TT sequences consistent with a lack of mutagenicity of the CPDs or (6–4)PPs at these sequences. This finding has been supported by experiments in which synthetic thymine–thymine CPDs have been shown to be poorly mutagenic using in vitro and in vivo studies (Banerjee et al. 1988; Gibbs and Lawrence 1993; Gentil et al. 1996). Surprisingly, in these experiments with synthetic CPDs, even the 5′TC and 5′TmC dimers were bypassed correctly by DNA polymerases 95–99% of the time (Horsfall et al. 1997) (Vu et al. 2006). Considering these data, how can we understand why such a large fraction of UVB-induced mutations are C to T transitions at dipyrimidine sites?

The low mutagenicity of 5′TT dimers has been largely explained by the discovery of specialized DNA polymerases that bypass these photolesions in a correct manner by incorporating two adenine bases across the lesion (also referred to earlier as the “A rule”). In mammals, this specialized, non-replicative DNA polymerase is the product of the *POLH* gene and is referred to as Pol eta (POLH) (Johnson et al. 1999; Johnson et al. 2000a, b). In humans, mutations of *POLH* are found in one form of xeroderma pigmentosum, the XP variant complementation group or XP-V (Masutani et al. 1999). Pol eta-deficient cells or mice have an increased mutation frequency when exposed to UV light and the patterns of mutations indicate that Pol eta contributes to correct bypass of UV dimers (Stary et al. 2003; Choi and Pfeifer 2005; Busuttil et al. 2008; Kanao et al. 2015). During DNA replication of a CPD, after replicative DNA polymerases become arrested at UV lesions, the trans-lesion synthesis is accomplished by POLH. There is considerable evidence to support the idea that POLH is chiefly responsible for correct CPD bypass in yeast and mammals (Kozmin et al. 2003; Choi and Pfeifer 2005; Yoon et al. 2009). In addition to POLH, the scaffold protein REV1 is also important in promoting correct bypass of CPD lesions as it works in conjunction with the lesion-bypass polymerases (Yoon et al. 2015). However, the contribution of translesion synthesis (TLS) polymerases to skin cancer avoidance is not straightforward. Whereas lack of POLH is clearly linked to skin cancer development (as seen in XP-V patients) and is generally thought to be due to increased UV-induced base substitution mutations, inactivation of the error-prone TLS DNA polymerase Pol theta (POLQ) also gives rise to increased incidence of skin cancer in mice. The authors of that study proposed that one important role of TLS polymerases is to prevent replication fork stalling and thus help to avoid formation of double-strand break-induced genomic rearrangements (Yoon et al. 2019).

One aspect of UV mutagenesis that is often overlooked is the substantial contribution of modified cytosine bases to the mutational processes. In mammalian cells, most of the DNA cytosine methylation events occur at 5′CG dinucleotide sequences. Simulated sunlight-induced mutagenesis in a heavily methylated mutational reporter gene is strongly targeted to methylated PymCG sites. These trinucleotides stand out as mutational hotspots with sunlight but not with 254 nm UVC radiation (You et al. 1999; You and Pfeifer 2001). Indeed, the sequence TmC may form more CPDs with solar irradiation compared to the commonly known TT dimers.

**Fig. 2:**
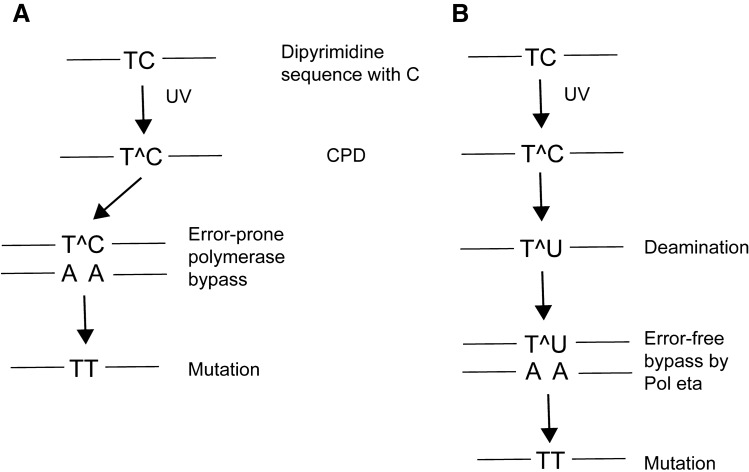
Pathways leading to UV mutagenesis at CPDs containing cytosine. **a** In the error-prone DNA synthesis pathway, a DNA polymerase bypasses the CPD by insertion of an incorrect adenine base across the cytosine leading to C to T mutations. **b** In the deamination-bypass pathway, the CPD that has formed at dipyrimidines containing cytosine undergoes hydrolytic deamination to uracil. DNA synthesis past uracil-containing dimers by DNA polymerase eta occurs in an error-free manner by incorporation of adenine across uracil. However, the mutation is fixed due to the deamination event. Note that a similar pathway may operate at CPDs containing 5-methylcytosine. In that case, deamination leads to the formation of thymine within the dimers followed by error-free Pol eta bypass of the lesion. UV-induced CC to TT mutations may arise from double cytosine or 5-methylcytosine deamination events

There are at least two possible mechanisms to explain the dominance of C to T transition mutations at dipyrimidines containing cytosine. The first one would invoke bypass of the dimer by a DNA polymerase that incorporates adenine (Fig. 2a). However, UV dimers at 5′TC sequences are in fact bypassed correctly by incorporation of 5′GA across the lesion by DNA polymerases, as first shown in *E. coli* (Horsfall et al. 1997). Also, in vitro synthesis past a 5-methylcytosine-containing cyclobutane pyrimidine dimer by yeast Pol eta is mostly non-mutagenic (Vu et al. 2006; Song et al. 2012). In yeast and mammalian cells, Pol eta has a high bypass fidelity at dimers formed at 5′TC and 5′CC sequences, whereas other DNA polymerases like Pol kappa, Pol zeta or Pol theta may carry out CPD bypass erroneously in vivo (Yu et al. 2001; Yoon et al. 2009, 2019). The lesion bypass of (6–4)PPs appears to be more complex and involves a two-step mechanism in which a Pol eta-related (*Y* family) DNA polymerase first inserts a base across the lesion and the extension from the inserted nucleotide is then carried out by DNA polymerase zeta (Johnson et al. 2000a, b; Akagi et al. 2019).

Second, a perhaps more likely mechanism of UV mutagenesis is based on the knowledge that cytosines within CPDs are prone to hydrolytic deamination (Setlow et al. 1965; Lemaire and Ruzsicska 1993). Within a CPD, the stabilization of the N4 amino group of cytosine by aromatic resonance is lost due to saturation of the 5,6‐double bond leading to enhanced reactivity of the base with a water molecule. No enzyme is known that would deaminate cytosine within pyrimidine dimers. After deamination has occurred, the dimer will contain uracil, and bypass of such lesions by Pol eta will lead to incorporation of adenine, which results in a C to T mutation, not because of erroneous lesion bypass but because of the deamination reaction (Fig. 2b). There is considerable evidence to support the relevance of CPD deamination in UV mutagenesis (Jiang and Taylor 1993; Tu et al. 1998; Lee and Pfeifer 2003; Takasawa et al. 2004; Vu et al. 2006; Song et al. 2011). Studies with synthetic lesions provided initial support for the deamination-bypass model (Jiang and Taylor 1993). Using direct analysis of deaminated dimers in cells, by which deamination of CPDs is assessed using consecutive photolyase reaction and uracil DNA glycosylase incision, it was shown that deamination occurs in a time-dependent manner and leads to the accumulation of substantial levels of deaminated cytosine-containing dimers (Tu et al. 1998). The deamination pathway is likely of the highest importance in slowly dividing cells (such as they are found in human skin) and in settings where repair of CPDs is relatively slow. There is also evidence that 5-methylcytosine, in particular at 5′TCG sites, can effectively deaminate to thymine (Cannistraro et al. 2015). This mechanism may further contribute to the selective UVB mutagenesis found at 5′-PymCG sites (You et al. 1999; Lee and Pfeifer 2003).

## Mapping of UV damage in the mammalian genome: effects of nucleosomes and transcription factors

The formation and repair of UV photoproducts along the mammalian genome are not random. Although (6–4) photoproducts are more frequent in nucleosomal linker DNA than in nucleosome core DNA, CPDs have no overall preference for linker or core regions (Mitchell et al. 1990). However, CPDs form with a 10 base pair periodicity consistent with rotational setting of the DNA on nucleosome core particles (Gale et al. 1987). Thus, the CPDs form preferentially where the DNA phosphate backbone is farthest away from the histone surface.

**Fig. 3:**
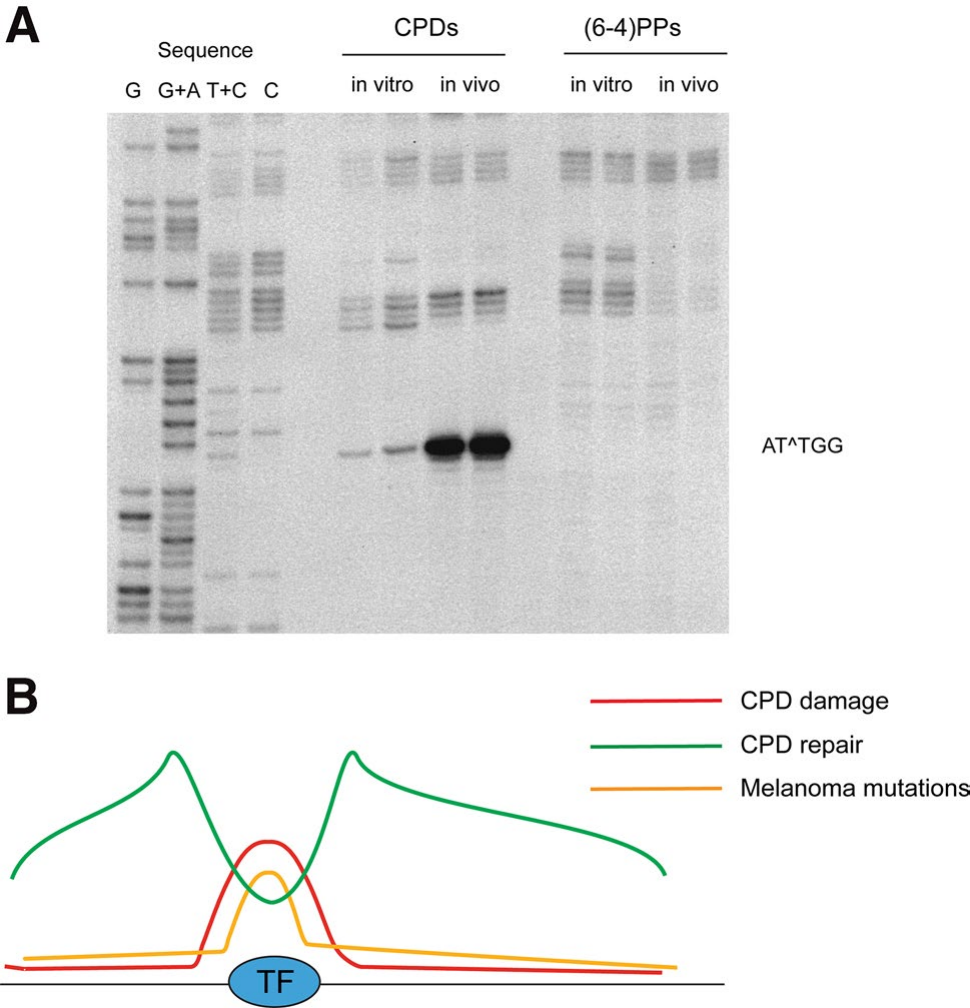
UV damage at transcription factor binding sites and melanoma mutations. **a** A region 100–150 base pairs upstream of the transcription start site of the *PCNA* gene acquires high levels of CPDs after UV irradiation of HeLa cells at a specific position containing a consensus binding site of the NFY transcription factor (5′ATTGG). CPDs and (6–4)PPs were mapped separately in this region (Tornaletti and Pfeifer 1995). Note that these CPDs at 5′TT sequences would not be mutagenic. **b** Schematic illustration of high levels of UV damage (CPDs) at transcription factor (TF) binding sites genome-wide, generally enhanced repair in open chromatin regions surrounding the TF but diminished repair at the TF binding site itself, and the resulting mutation hotspots targeted to such binding sites in melanoma skin tumor genomes (Sabarinathan et al. 2016; Elliott et al. 2018)

In earlier work on UV damage, it was possible to precisely localize UV-induced DNA damage, in particular CPDs, to specific positions in the mammalian genome. The ligation-mediated polymerase chain reaction (LMPCR) technique can be used for base resolution mapping of such damage (Pfeifer et al. 1991, 1992). For CPD mapping, the method is based on cleavage of genomic DNA at CPD sites using the CPD-specific T4 endonuclease V, a DNA glycosylase that incises DNA at CPDs to create a single-strand break. The dimerized pyrimidine bases remaining at the break are then repaired using *E. coli* photolyase and long-wave UVA light. These treatments result in clean DNA strand breaks with a 5′-phosphate group at the initial CPD site. To analyze CPDs within specific genes, gene-specific primers are used for primer extension, followed by ligation of a linker, PCR amplification and sequencing of the fragments by gel electrophoresis. The method is sensitive enough to map CPDs at frequencies of less than one CPD per 10–20 kilobases of DNA. Using this approach, it is possible to identify genomic sites with particularly high levels of CPDs accumulating after UVB or UVC irradiation. Surprisingly, the most severely damaged DNA sequences were found in gene promoters (see Fig. 3a for an example) (Pfeifer et al. 1992; Tornaletti and Pfeifer 1995). They coincided with binding sites of several sequence-specific transcription factors, which apparently make the DNA more susceptible to dimer formation by introducing a favorable structural distortion into the DNA double helix. Indeed, stacked dimers with reduced inter-base distances can form with lower energy excitation transitions in curved and highly distorted DNA structures (Ramazanov et al. 2015). In contrast to these damage hotspots, other transcription factor-bound sites sustained much less CPD or (6–4)PP damage in cells compared to naked DNA, presumably because the bound factors were associated with a more rigid DNA structure that is incompatible with dimerization of the adjacent DNA bases (Tornaletti and Pfeifer 1995).

The high sensitivity of this method also allowed the analysis of DNA repair kinetics for CPDs at the DNA sequence level (Gao et al. 1994; Tornaletti and Pfeifer 1994). These studies uncovered that slow DNA repair occurs at specific sites in the *TP53* tumor suppressor gene, at sequences that are also frequently mutated in nonmelanoma skin cancers (Tornaletti and Pfeifer 1994). It was possible to verify the previously known rapid repair of transcribed DNA strands relative to non-transcribed DNA strands (Mellon et al. 1987) at high resolution. Furthermore, this approach identified regions of particularly efficient DNA repair near the transcription initiation sites of several genes (Tu et al. 1996a, b; Tommasi et al. 2000) suggesting that open chromatin structure and transcription may facilitate nucleotide excision repair of CPDs. In contrast, excision repair of the upstream gene promoter regions occupied by transcription factor complexes was notably slow (Gao et al. 1994; Tu et al. 1996a, b; Tommasi et al. 2000). Consistent with these findings, genome sequencing studies of melanomas exhibiting a prominent UV-related mutation signature showed a strong enrichment of promoter mutations and mutations at transcription factor binding sites (Perera et al. 2016; Poulos et al. 2016; Sabarinathan et al. 2016).

When different UV radiation sources were used for irradiation of human keratinocytes, the positions of the modified DNA base 5-methylcytosine at methylated CpG sequences that have a 5′ neighboring pyrimidine (5′TmCG and 5′CmCG) were associated with particularly high levels of CPDs, but interestingly, only when sunlight was used for irradiation (Tommasi et al. 1997). In skin cancers, mutations in the *TP53* gene are frequent at these trinucleotide positions. In fact, the distribution of CPDs induced by sunlight gave a good match with the mutations found in human skin tumors (Tommasi et al. 1997). The reasons for the increased CPD formation at methylated cytosines are still not entirely clear. First of all, 5-methylcytosine has an absorption maximum that is shifted toward longer wavelength relative to cytosine (You et al. 1999). With 5-methylcytosine, there is a tenfold increase of the fluorescence lifetime, making excited state reactions more probable (Sharonov et al. 2003). It also seems that the presence of the methyl group on cytosine affects the sugar puckering, thereby enhancing DNA duplex conformations that are more prone to CPD formation (Banyasz et al. 2016). In addition to 5-methylcytosine, its oxidized derivative 5-hydroxymethylcytosine also effectively undergoes CPD formation (Kim et al. 2013) and so do dipyrimidines containing 5-formylcytosine or 5-carboxylcytosine (Sang-In Kim and Gerd P. Pfeifer, unpublished observations).

Instead of detecting UV damage in specific genes and even at the base level of resolution, the focus of these UV damage mapping studies has now shifted toward mapping the damage genome-wide and preferably at every base position. Although still associated with high sequencing costs, there are now several methods available to accomplish these goals. As one example, to achieve medium-resolution mapping at a genome-wide coverage, one can use antibodies against CPDs to immuno-precipitate the UV-irradiated DNA. The collected CPD-containing DNA fragments are then prepared as a sequencing library and sequenced on high-throughput DNA sequencing systems. The level of resolution achieved with this approach is similar to that obtained in ChIP-sequencing studies of chromatin-bound proteins and can reveal genomic regions that are highly susceptible to damage within a sequence window of 100 to 200 base pairs (Garcia-Nieto et al. 2017).

To achieve single-base resolution for UV damage distribution genome-wide, more complex technologies are required. In a method called CPD-seq, DNA is first sheared randomly by sonication and is then ligated to adapters on which the 3′ ends are blocked with a dideoxynucleotide. The CPDs are then specifically incised using T4 endonuclease V and a random hexamer adapter is ligated into these break positions followed by DNA sequencing (Mao et al. 2016, 2017). Interestingly, this whole genome mapping of CPDs in irradiated human cells showed enhanced damage formation at binding sites of the ETS transcription factor (consensus sequence 5′CTTCC) and other transcription factors (Mao et al. 2018). A similar result of enhanced CPD formation at ETS binding sites was reported in two other studies (Elliott et al. 2018; Premi et al. 2019). This result is conceptually similar to what has been observed previously at several other transcription factor binding sites using single gene analysis (Pfeifer et al. 1992; Tornaletti and Pfeifer 1995). Enhanced CPD formation at ETS transcription factor binding sites correlated globally with enhanced mutation frequencies near the same DNA sequences in melanoma (Fredriksson et al. 2017; Elliott et al. 2018; Mao et al. 2018) (Fig. 3). These mutations did not affect gene expression in a major way indicating that the mutations are for the most part localized DNA damage-driven passenger events in the melanoma genomes. The situation is different, however, for the telomerase reverse-transcriptase (*TERT*) gene promoter in human melanoma. Here, UV signature C to T mutations create de novo binding sites for ETS transcription factors (Horn et al. 2013; Huang et al. 2013), which should result in upregulation of *TERT* expression, an event that is expected to promote cell immortalization.

## Repair of dimeric DNA photoproducts

The pioneering work of James Cleaver indicated that the sun-sensitive and cancer-prone human syndrome xeroderma pigmentosum (XP) is characterized by a defect in DNA repair or processing of UV damage (Cleaver 1969). XP patients are extremely sun-sensitive and have a up to 1000-fold increase in the incidence of skin cancers, both melanoma and non-melanoma (Hanawalt and Sarasin 1986). Dimerized base- and bulky adduct-containing DNA lesions such as UV photoproducts are generally repaired by the nucleotide excision repair (NER) pathway. These data suggested that pyrimidine dimers are responsible for the increased tumor susceptibility of these patients. CPDs are repaired much more slowly than (6–4)PPs, perhaps due to their lower propensity to destabilize the DNA double helix (Mitchell et al. 1985). Defects in base excision repair, which would eliminate other types of DNA damage such as oxidized guanines, have generally not been associated with skin cancer prone syndromes in humans although the OGG1 knockout mouse, which is deficient in repair of 8-oxo-dG, has an increased susceptibility for UVB-induced skin cancer formation (Kunisada et al. 2005).

The individual steps of nucleotide excision repair are well understood (Marteijn et al. 2014; Spivak 2015; Araujo and Kuraoka 2019). In brief, during global genome repair, the dimeric UV photoproducts are recognized by the XPC/RAD23B protein complex, which may be aided in chromatin by the UV-damaged DNA-binding protein (UV-DDB) complex exhibiting ubiquitin ligase activity. The UV-DDB complex is most important for the recognition and repair of CPDs within a chromatin environment, whereas (6–4)PPs may be recognized directly by the XPC complex (Gsell et al. 2020). In a particularly effective sub-pathway of NER called transcription-coupled repair (TCR), the UV lesions are repaired rapidly on the transcribed strand of active genes. In this case, recognition of the lesion is accomplished by the stalling of RNA polymerase II at the damage site. After stalling, the transcription–repair coupling factor CSB (mutated in Cockayne syndrome type B), in conjunction with CSA (mutated in Cockayne syndrome type A), aids in the backtracking of RNA polymerase II to make the UV lesions accessible to NER (Mullenders 2015; Pani and Nudler 2017). Following lesion recognition in either one of the two sub-pathways, the NER factors XPA and TFIIH, along with replication protein A (RPA), open and stabilize the DNA helix at the damage site and make it accessible to the lesion cleavage endonucleases XPF/ERCC1, which cleaves the lesion-containing strand of the DNA on the 5′ side of the lesion and XPG, which cleaves the same strand on the 3′ side of the lesion (Araujo and Kuraoka 2019). After this dual cleavage, a DNA fragment of 24–32 nucleotides is released that contains the dimerized pyrimidine. The resulting gap is then filled by replicative DNA polymerases and is eventually sealed by DNA ligases. NER seems to be the major and perhaps the only DNA repair pathway that deals with UV-induced pyrimidine dimers in mammalian cells.

In addition to transcription, chromatin structure and nucleosome occupancy may regulate the accessibility and repair of UV lesions in the genome. A number of studies have shown that positioned nucleosomes inhibit DNA repair of CPDs. For details, the reader is referred to earlier articles and reviews of this subject (Pfeifer 1997; Wellinger and Thoma 1997; Mao et al. 2017). Repair of UV photoproducts in a chromatin or nucleosomal environment is highly complex and the proposed mechanisms have recently been summarized (Gsell et al. 2020). Repair of CPDs involves chromatin remodeling events as well as facilitation of individual steps by histone lysine methyltransferases including the H3K79 methyltransferase DOT1L and the H3K4 and H3K36 methyltransferases ASH1L and NSD2, which may generate docking sites for reader proteins during the repair reactions (Gsell et al. 2020). However, individual details of the steps involved are not exactly known, the published data do not always agree with each other, and much remains to be learned about how lesion accessibility and repair in chromatin are regulated.

The differential, chromatin-mediated DNA damage and repair activities in vivo (Adar et al. 2016; Mao et al. 2016, 2017) are reflected in nonrandom patterns of mutagenesis, for example in melanoma genomes (Schuster-Bockler and Lehner 2012; Woo and Li 2012; Liu et al. 2013; Mao et al. 2020), where regions of higher UV damage formation or slower repair are characterized by increased frequencies of mutations. At the level of positioned nucleosomes, this is strikingly apparent by a mutational pattern in melanomas where the peaks of UV-signature mutations at dipyrimidines occur with a periodicity of 10 bp at positions where the minor groove of the DNA is facing away from the nucleosome core particle (Brown et al. 2018; Pich et al. 2018).

**Fig. 4:**
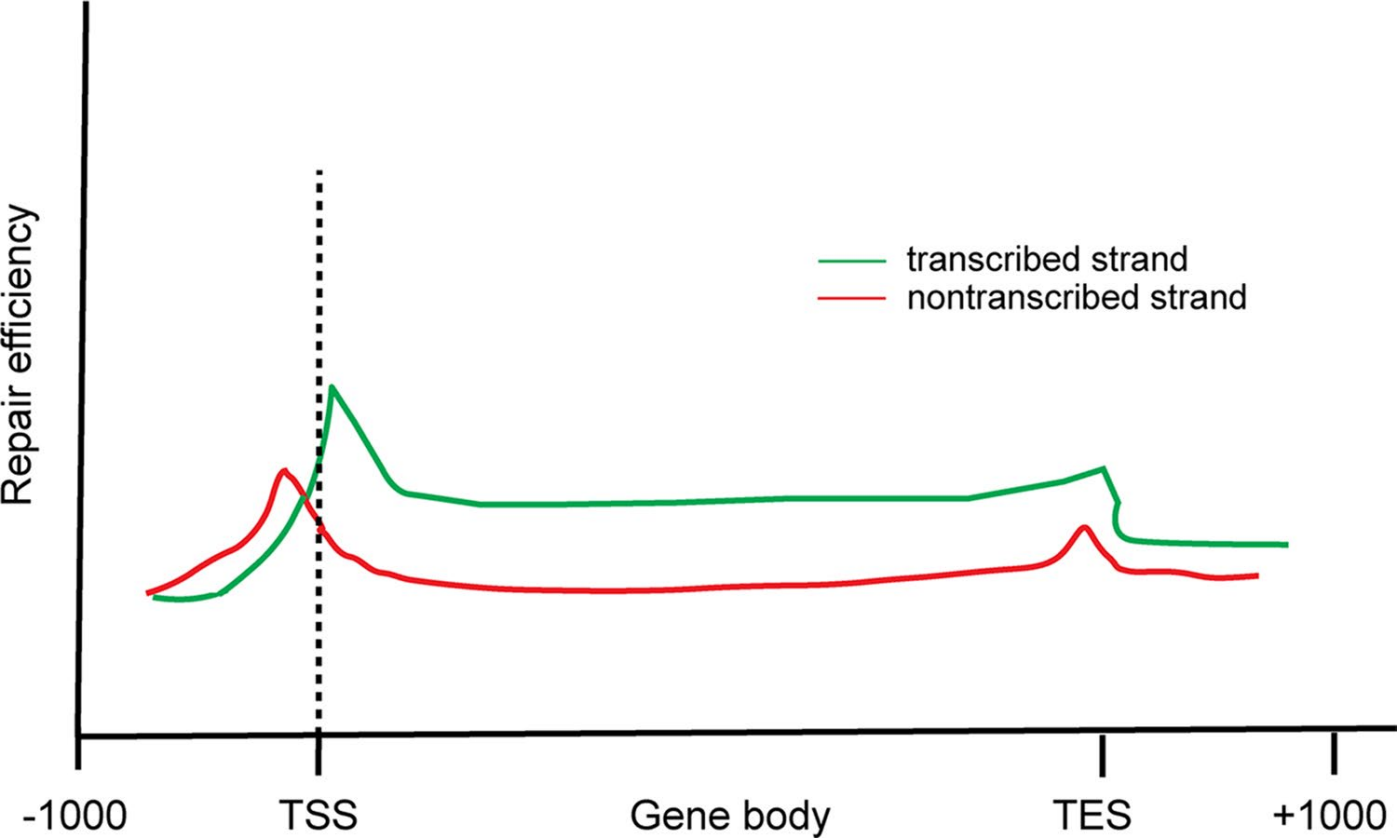
Genome-wide maps of nucleotide excision repair of CPDs. The XR-seq method was used to obtain genome-wide single nucleotide resolution maps of repair of CPDs. The figure shows a schematic illustration of the data obtained by Hu et al. (2015) for excision repair of CPDs in normal human fibroblasts. The graphs shown are average profiles for all UCSC reference genes. Note the more extensive repair of the transcribed DNA strand relative to the nontranscribed strand, the fast repair near the transcription start sites (TSS) and a slightly enhanced repair near the transcription end sites (TES). The faster repair of the nontranscribed strand upstream of the TSS is thought to be due to divergent transcription emanating from the promoters, which is a common feature of mammalian cells

One recently developed method to map DNA damage and repair at single-base resolution is referred to as excision repair sequencing or XR-seq (Hu et al. 2015). In this approach, the excision of the UV-induced lesions occurs in vivo. The excised ~ 26 to 27 bp long DNA fragments (in humans) are isolated by immunoprecipitation with anti-TFIIH antibodies (a nucleotide excision repair protein complex, see above) and are then sequenced using high-throughput sequencing methods (Hu et al. 2015; Adar et al. 2016). This method gives an indication of UV damage repair activity at all sites in the genome and integrates the level of UV damage at particular sites and its repair efficiency. When UV photoproduct-specific antibodies are used to collect the fragments, the repair of CPDs or (6–4) photoproducts can be analyzed separately (Adar et al. 2016). These genome-wide DNA repair maps have shown that active chromatin regions, as indicated by the presence of DNAseI hypersensitive sites and the presence of active chromatin marks including H3K4 trimethylation and H3K27 acetylation, are repaired more rapidly than DNA regions embedded in heterochromatin (Adar et al. 2016; Hu et al. 2017). These studies have confirmed at much higher resolution the previously known preference for repair of the transcribed DNA strands relative to non-transcribed strands or genomic regions without genes (Hu et al. 2015) (see, Fig. 4).

## Ultraviolet radiation and skin cancer risk

### Basal and squamous cell carcinoma

The incidence of non-melanoma skin cancers derived from epidermal cells (both basal and squamous cell carcinoma) increases with age (Albert et al. 2019). There has been a quite dramatic increase of these types of cancers in many parts of the world recently (Perry et al. 2017). It has long been known that basal and squamous cell skin cancers are strongly linked to UV exposure from the sun. The risk is highest in white (Caucasian) populations and is UV dose-dependent (Xiang et al. 2014).

### Melanoma

Due to its propensity to metastasize, sometimes even early during the tumor evolution process, melanoma is the most dangerous form of human skin cancer. Its incidence has shown a steady increase in many countries in recent decades (Gilchrest et al. 1999; Dimitriou et al. 2018). Compared to basal or squamous cell carcinomas, the epidemiological evidence linking melanoma with sunlight exposure has always been less compelling. Some studies have linked early sun exposure episodes in childhood, in particular strong exposures associated with sunburn, with a higher probability of developing the disease later in life (Pho et al. 2006; Leiter and Garbe 2008). Other studies have suggested that the UVA component of sunlight is particularly relevant for melanoma risk (Setlow 1974; Langford et al. 1998; Moan et al. 1999; Woodhead et al. 1999; Wang et al. 2001). Such epidemiological studies are sometimes difficult to interpret because the results may be biased by an inability of study participants to precisely remember and quantify such early life exposures as well as by many other factors.

In animal studies, both UVB and UVA radiation sources can induce squamous-cell carcinoma in mice (de Gruijl et al. 1993) (de Gruijl 2002). Using simulated sunlight, it was estimated that UVA may contribute 10–20% and UVB 80–90% of the tumor-inducing dose of sunlight (de Laat et al. 1997). The ability of UVA to induce melanoma in experimental animal models has been unclear. Earlier it was reported that UVA produces melanoma-like lesions in certain fish species (Setlow et al. 1993) and in marsupials (Ley 1997). However, other studies did not confirm melanoma formation by UVA in either a *Xiphophorus* fish model (Mitchell et al. 2010) or in mice (De Fabo et al. 2004). A more recent study has again concluded that UVA can induce melanoma in a mouse model and that melanin is required for this process (Noonan et al. 2012).

Of concern, high-energy UVA tanning lamps, even those emitting mostly between 340 and 400 nm (also referred to as UVA1 wavelengths), may have carcinogenic potential as shown in epidemiological studies linking these practices to melanoma incidence (Autier et al. 1994; Lazovich et al. 2010). However, a causal link between tanning bed exposure and melanoma risk is still controversial (Suppa and Gandini 2019; Reichrath et al. 2020). Use of UVB sun blockers that do not block UVA increases the exposure of a person to much higher doses of UVA because they can spend more time in the sun without experiencing painful UVB-induced sunburn.

Regarding the role of UVA in skin cancer, it is known that this wavelength induces relatively low levels of oxidative DNA damage but may create certain levels of CPDs (Douki et al. 2003; Ikehata et al. 2008; Pfeifer and Besaratinia 2012; Schuch et al. 2017). A role of cellular photosensitization reactions mediated by chromophores exposed to UVA has been discussed (Cadet et al. 2012, 2015). However, the relevance of these mechanisms for skin cancer induction is still not well proven. Quite surprisingly, a recent study showed that CPDs can also form in the dark, several hours after initial irradiation of melanocytes with a UVA light source (Premi et al. 2015). This reaction is thought to be mediated through the formation of reactive oxygen species in the UVA-irradiated cells, with one of them, peroxynitrite, being able to excite melanin derivatives to a triplet state. This triplet state energy is then transferred to DNA to induce CPD formation in the dark (Premi et al. 2015). The exact details of this novel mechanism are still unclear. The described physicochemical process may suggest that the contribution of UVA to melanoma may be more significant than previously thought and that the pathway operates through CPDs as the major mutagenic lesion. Furthermore, UVA may induce CPDs in DNA directly via a photochemical mechanism (Jiang et al. 2009; Mouret et al. 2010). However, the relevance of these mechanisms for UVA-mediated melanoma formation in human remains poorly defined.

## Mutations in skin cancer genomes

The first UV-specific mutations in nonmelanoma skin cancers were described for the *TP53* tumor suppressor gene (Brash et al. 1991; Ziegler et al. 1993). This gene is not very commonly mutated in melanoma. Data from cancer genome sequencing have provided key information on the exposures that may be relevant for cancer formation in humans (Pfeifer 2015). Throughout the past decade, high-throughput DNA sequencing has been used increasingly to more clearly define the mutational patterns and signatures of skin cancer genomes. When this work initially began by sequencing a few hundred protein kinase genes, the authors noticed a strong accumulation of C to T transition mutations in human melanoma (Greenman et al. 2007). This type of work greatly accelerated with a whole genome sequencing study of a melanoma cell line (Pleasance et al. 2010). By comparing the mutation data from this cell line with germline lymphoblastoid DNA from the same patient, the authors identified more than 30,000 base substitution mutations in the melanoma-derived cell line. The most frequent types of mutations were C to T mutations, which represented more than two-thirds of all mutations in the tumor sample. Quite consistent with the mutation patterns commonly seen in UVB-irradiated cells using, for example, mutation reporter gene analysis (You et al. 2001), more than 90% of these C to T transition mutations were found within a dipyrimidine sequence context, mostly 5′TC and 5′CC. One of the most well-known UVB mutation signatures consists of tandem CC to TT mutations, and this double-base substitution mutation was strongly elevated in this melanoma genome. These CC to TT double substitutions showed a high frequency in the otherwise rare DNA sequence context of 5′CCG (10%), which are methylated to contain 5mC in most compartments of the human genome. When the UV-related transition mutations were analyzed with respect to strand specificity in expressed genes, the authors found that there were more such mutations on the non-transcribed DNA strand consistent with transcription-coupled DNA repair (Pleasance et al. 2010). The frequent occurrence of the UV signature mutations in cutaneous melanomas has since been confirmed in several subsequent studies (Hodis et al. 2012; Trucco et al. 2019). Melanomas from sun-exposed body sites had more UV-related C to T signature mutations compared to melanomas arising in sun-protected areas (Krauthammer et al. 2012). This UV signature is already present in melanoma precursor lesions (Shain et al. 2015).

Genomic profiling of 293 human skin basal cell carcinomas uncovered a strong UV damage-related mutational signature in almost all cases (Bonilla et al. 2016). The majority of squamous cell carcinomas of the skin also carry a strong UV signature and a large number of such mutations (Durinck et al. 2011; Pickering et al. 2014; Inman et al. 2018; Mueller et al. 2019). Merkel cell carcinoma is a rare type of neuroendocrine skin tumor. Up to 80% of these cases carry the Merkel cell polyomavirus (MCV) and have low mutation rates. However, MCV-negative cases had a high mutation burden and a UV-type signature mutation pattern (Wong et al. 2015). Of relevance for the keratinocyte-derived skin cancer types, the typical UV signature mutation pattern is already present in cancer-free sun-exposed skin. This had first been noted in sun-exposed human skin that contains clonal patches of *TP53* mutated keratinocytes (Jonason et al. 1996). Martincorena et al. took small biopsies from four individuals who underwent eyelid surgery (Martincorena et al. 2015). They sequenced 74 cancer-relevant genes and the entire genome of one biopsy. These specimens exhibited clear characteristics of mutational signatures resulting from sun exposure including C to T mutations at 5′TC and 5′CC dinucleotides and also CC to TT mutations. G to T mutations potentially resulting from oxidative damage to guanine were also detected. Considering the ratios of non-synonymous to synonymous mutations, the authors identified five potential driver genes that were positively selected and were associated with clonal cell expansions, *NOTCH1*, *NOTCH2*, *NOTCH3*, *TP53* and *FAT1*. About 20% of normal skin cells were estimated to have *NOTCH1* mutations, a mutation that is commonly found in squamous cell carcinomas of the skin (Wang et al. 2011; Pickering et al. 2014). Even though some cell clones contained two or three driver mutations, no malignancy was detected raising the interesting question of how many driver mutations are required for skin cancer to arise, or if other non-mutagenic but tumor-promoting pathways also need to be engaged. Similar results were obtained by Yizhak et al. who observed that sun-exposed skin had more mutations than nonexposed skin and contained the highest number of mutations of any normal tissue analyzed. The UV signature dipyrimidine mutations were found in 62 of the 67 sun-exposed skin samples, and *NOTCH1* and *TP53* were commonly mutated (Yizhak et al. 2019).

It is remarkable, however, that skin tumor genomes (melanoma and nonmelanoma) contain overall relatively low contributions from G to T transversion mutations, a mutational event that would be expected from oxidative DNA damage to guanine. These findings reaffirm the notion that most sunlight-induced mutations in human skin can be traced back to dimeric DNA photoproducts.

## Driver mutations in human skin cancer

Basal and squamous cell carcinoma: Basal cell carcinomas of the skin carry one of the highest load of mutations of any tumor type (Jayaraman et al. 2014; Bonilla et al. 2016). Sequence analysis of these tumors identified common mutations in the sonic hedgehog pathway. The *PTCH1* gene, encoding a receptor for secreted hedgehog ligands, was mutated in about 75% of the cases (Jayaraman et al. 2014; Bonilla et al. 2016) and had over 60% UV signature mutations. Also, frequently mutated was the *TP53* tumor suppressor gene, which carried mutations in about 60% of the basal cell tumors. Other driver genes were also mutated but at a lower frequency. In squamous cell carcinoma, the likely tumor-driving genes *NOTCH1*, *TP53*, *FAT1* and several other were the most frequently mutated ones (Pickering et al. 2014; Inman et al. 2018).

Melanoma: The *TP53* tumor suppressor gene, which is the most frequently mutated gene in most human cancer types (Hainaut and Pfeifer 2016), is less commonly mutated in melanomas. A first clue to identifying tumor-driving mutations in melanoma came from the existence of germline mutations in the CDK inhibitor gene *CDKN2A* (also known as *p16*) (Kamb et al. 1994). Rare familial cases of melanoma often contain mutations in *CDKN2A*. Such mutations are also seen in sporadic, non-inherited cases of melanoma supporting a role of this gene as a driver gene and melanoma suppressor (Hocker and Tsao 2007). Many of the *CDKN2A* gene mutations are C to T transitions at dipyrimidine sites. One of the most frequently mutated genes in melanoma is *BRAF* encoding a protein kinase in the RAS signaling pathway. However, mutations in BRAF at codon 600 are not typically UVB signature mutations although a potential sunlight-mediated origin of these mutations is theoretically possible (Thomas et al. 2006; Besaratinia and Pfeifer 2008). More likely, the preponderance of this mutation is due to strong selection for a specific mutant form of the kinase. Other frequently mutated genes in melanoma include the genes *GNAQ* and *GNA11*, which encode heterotrimeric G protein subunits, as observed in uveal melanomas (Van Raamsdonk et al. 2010). These mutations may be induced by UVB irradiation as they are true C to T signature mutations at dipyrimidine sites (Besaratinia and Pfeifer 2011).

## Concluding remarks

UV radiation from the sun produces various types of DNA lesions. Considering the narrow UV waveband window of sunlight that reaches the surface of the earth and can effectively damage DNA, it may be concluded that the cyclobutane pyrimidine dimers are the type of DNA damage most relevant for human exposure. In the terrestrial UVB range, this lesion is much more abundant than the (6–4) photoproducts or oxidized base damage. There is also a characteristic mutational signature associated with CPDs in experimental systems, and it is this signature that is highly prevalent in both nonmelanoma and melanoma skin tumor genomes. CPDs are not only abundant but also relatively persistent DNA lesions owing to their slow repair by nucleotide excision repair. Specialized DNA polymerases have evolved to carry out a mostly non-mutagenic bypass of CPDs. These polymerases traverse CPDs with all dipyrimidine combinations with incorporation of the correct base-pairing nucleotides. However, the polymerases are ineffective in preventing a chemical reaction that occurs before DNA replication, the deamination of cytosines or 5-methylcytosines within CPDs. This deamination reaction very likely is the key mechanistic step in UV mutagenesis. Future work is needed to further confirm this mechanism and perhaps to find ways to prevent it from occurring in sun-exposed skin.

## Abbreviations

UV light: Ultraviolet light
CPD: Cyclobutane pyrimidine dimer
(6–4)PP: Pyrimidine (6–4) pyrimidone photoproduct
8-oxo-dG: 8-Oxo-7,8-dihydro-2′-deoxyguanosine
XP: Xeroderma pigmentosum
NER: Nucleotide excision repair
5mC: 5-Methylcytosine
TLS: Translesion synthesis
